# Necrosis of Nearly the Whole of the Lower Jaw—Removal of the Dead Bone, Including One Condyle—Recovery with Perfect Movement of the Jaw

**Published:** 1870-02

**Authors:** 


### ARTICLE VI.

### *Necrosis of Nearly the Whole of the Lower Jaw—Removal of the Dead Bone, Including one Condyle—Recovery with Perfect Movement of the Jaw.*

(Under the care of Mr. Christopher Heath).

Egbert H., aged 22 from Aylesbury, was sent to Mr. Heath by Mr. Ceely with necrosis of the lower jaw.

In August, 1868, he had typhus fever in Walsall Union, and during the attack the face became swollen, and discharged both externally and into the mouth. His teeth were all loosened, but none were extracted. In December he was passed on to Aylesbury, and came under Mr. Ceely's care.

On February 24, 1869, patient was admitted into, University College Hospital under Mr. Heath's care. The right side of the lower jaw was immensely swollen, and two inches below the angle was a sinus through which a probe passed up towards the base. Another sinus existed below the right canine tooth, and there had been a third below the left angle which was now closed. The teeth were all more or less loose, and there were several openings in the gums, from which a most offensive discharge passed into the mouth. The man was well nourished and otherwise in good health, though he had when a child suffered from hip disease. On the day of admission, under chloroform, Mr. Heath extracted the molar teeth of the right side which were loose, and, having divided the gum, extracted a very large sequestrum, comprisng the right side of the body of the jaw from the canine tooth to the angle, and containing the mental foramen. The hæmorrhage was very free, but was checked by plugging the shell of new bone from which the seqеstrum was taken. The plugs were removed on the second day, and the mouth syringed out daily with disinfecting lotion.

On March 3, 1869, under chloroform, Mr. Heath cleared out some small fragments of necrosed bone left in the right angle of the jaw, and then proceeded to remove the necrosed portion on the left side, which extended as far as the second molar tooth. Mr. Heath attempted to save the incisor teeth it appearing at first that the alveolus of that part of the jaw was not involved. It proved, however, that the disease had affected the whole thickness of the bone, and the teeth were necessarily sacrificed. Upon removal of the sequestrum there was left a complete framework of new bone, with a deep groove extending from the right angle (which was quite hollowed out) to the second molar tooth of the left side. The mouth bled freely, but this was checked as before by stuffing with lint. The patient made a good recovery, and was able to return to the country in a week, the discharge having almost entirely ceased, and there being a deep groove in the new structure of the jaw from which the sequestrum had been extracted.

On June 16 the patient returned, there being a portion of diseased bone on the right side. This Mr. Heath extracted, under chloroform, with some diffculty through the mouth, when it was found to include the angle and a great part of the ramus of the jaw. From this operation also the patient made a speedy recovery, and returned to the country, and was not seen again by Mr. Heath until October, when he returned with yet more necrosis, involving the remainder of the right ramus. This was removed with difficulty on October 30, and the man has not since suffered from pain or discharge, so that it seems that the whole of the dead bone has now been taken away.

Perhaps the most singular feature in this case is the fact that the man has now (December) as perfect movement of the jaw as if no disease had existed, notwithstanding that at the last operation the whole of the right condyle was removed entire with about a third of the ramus. The repair has, in fact, been as complete as possible. When we saw the patient five weeks after the last operation, there was some fulness and prominence about the right angle of the jaw, and when the mouth was widely opened the lower jaw was drawn slightly to the right side ; but otherwise all the jaw movements were perfectly performed without any pain or inconvenience, a deep groove in the gum, reaching from the right angle to the second left molar, alone remaining to show the former seat of such extensive disease.—*London Med. Times and Gazette.*

---

